# Comparison of the Effects of Rocuronium Bromide and Cisatracurium Besylate on Intubating Conditions and Haemodynamic Response

**DOI:** 10.7759/cureus.57878

**Published:** 2024-04-08

**Authors:** Parvati S, Jayashree Sen

**Affiliations:** 1 Anaesthesia, Ananthapuri Hospital and Research Institute, Trivandrum, IND; 2 Anaesthesia, Datta Meghe Institute of Higher Education and Research, Wardha, IND

**Keywords:** haemodynamic stability, adverse effect, intubating condition, onset of action, neuromuscular blocker, rocuronium, cisatracurium

## Abstract

Background

General anaesthesia (GA) is predominantly important for conducting tracheal intubation; it should be quick and precise, having a prudent performance. It is preferable to use a neuromuscular blocking drug, which ideally should be highly potent, with a rapid onset and a short duration clinical effect in order to prevent the development of hypoxia during laryngoscopy and tracheal intubation and also avoid any changes in haemodynamics caused by the release of histamine, ganglion block, and anti-muscarinic actions. The non-depolarizing muscle relaxants rocuronium and cisatracurium don't have any noticeable independent side effects when used within the recommended dosage levels.

Aim

The aim was to compare the clinical efficacy of rocuronium bromide and cisatracurium besylate with respect to their property as muscle relaxants in producing favourable intubating conditions and to assess their haemodynamic stability. The objectives of the study were to evaluate the onset of action and any undesirable effects.

Methods

Between the ages of 20 to 60 years, 60 patients of either gender, divided randomly into groups of 30 each, of American Society of Anesthesiologists (ASA) physical status classification I and II, were put for elective surgical procedures to be done under general anaesthesia. Patients were given 0.6 mg kg-1 IV of rocuronium in Group R and 0.15 mg kg-1 IV of cisatracurium in Group C. After injecting the muscle relaxants, parameters were measured 60, 90, 120, 150, 180, 240, and 300 seconds later.

Result

Demographical variables like age, gender, and ASA physical status of the two groups were analogous. Group R had good to excellent/favourable intubating conditions by 90 seconds, and Group C by 240 seconds with comparable haemodynamic stability. The onset of action was significantly faster in Group R (92 ± 7.61 seconds) than in Group C (188 ± 40.88 seconds).

Conclusion

Rocuronium produced favourable intubating conditions having good haemodynamic stability and a statistically significant (p < 0.00001) faster onset of action in comparison to cisatracurium.

## Introduction

General anaesthesia (GA), which can be caused by a variety of anaesthetic drugs, is characterized by amnesia, controlled reversible unconsciousness, absence of pain perception throughout the body, and varying degrees of muscle relaxation that can lead to apnoea or respiratory depression. To maintain adequate gas exchange, endotracheal intubation must be performed in a quick, precise, and prudent manner. To prevent hypoxia during laryngoscopy and tracheal intubation, a rapid-onset neuromuscular block is preferred. A neuromuscular blocking agent's (NMBA's) ideal characteristics should be high potency, fast onset, and brief clinical duration without any alterations in haemodynamic status. Effects of histamine release, anti-muscarinic activities, and ganglion blocking are responsible for changes in the haemodynamic state [1,2].

NMBAs are of two types: depolarizing and non-depolarizing. All NMBAs and muscle relaxants are quaternary ammonium compounds, identical to acetylcholine (ACH) structurally. As a result, all muscle relaxants imitate ACH and derive their paralyzing effects from it. The neuromuscular blocking substances primarily prevent nerve impulse transmission at the neuromuscular junction. Much effort has been put forth over a long period of time to create NMBAs with a quick onset and a period of action of short duration [3]. As a result, the non-depolarizing NMBAs cisatracurium and rocuronium, which have an intermediate duration of action, were introduced into clinical practice [4,5]. Earlier NMBAs vecuronium and atracurium were eventually superseded by rocuronium in 1992 and cisatracurium in 1995. Cisatracurium is a benzylisoquinoline that is relatively new. Its excretion from the body is through an organ-independent Hoffmann elimination process, whereby bromamide is degraded to the metabolites laudanosine and monoquaternary acrylate [6]. Contrary to atracurium, cisatracurium does not cause histamine to be released from the mast cells in the clinical range. This release of histamine from mast cells can result in peripheral vasodilation, bronchospasm and skin flushing as well as hypotension. Cisatracurium, a non-depolarizing, intermediate-duration neuromuscular blocking drug is safe, with excellent cardiovascular stability. Cisatracurium does not modify blood pressure or heart rate or create any autonomous motion after administration even at dosages as high as 8 × ED95.

Rocuronium, a monoquaternary compound, an analogue of vecuronium aminosteroid, is a non-depolarizing, intermediate-duration neuromuscular blocking drug, which is relatively of modest potency with a quick onset of action. It is largely eliminated via the liver and up to 20% of it can be excreted unaltered in urine [7]. The sole metabolite of rocuronium, 17-desacetyl rocuronium, has only 5-10% action of the parent compound and has no ability to discharge histamine [8]. Therefore, a prospective, randomized comparative study on the non-depolarizing muscle relaxants following a single injection of 2 × ED95 has been undertaken with the aim of comparing the clinical efficacy of rocuronium bromide and cisatracurium besylate for a favourable intubating condition by the correlation of the variables such as the degree of jaw relaxation and vocal cord immobility and to assess their haemodynamic stability. The objectives were to evaluate the onset of action and any related unfavourable effects.

## Materials and methods

Ethics consideration, study design, and participants

Once approval from the Institutional Ethics Committee, Datta Meghe Institute of Medical Sciences, Ref.No.DMIMS(DU)/IEC/2018-19/7377, and patients' informed written consents were obtained, this prospective, randomised, observational study was conducted within the patients admitted to a rural hospital medical college, namely, Datta Meghe Institute of Medical Sciences, in Maharashtra, India. A total of 60 patients of either gender, between the ages of 20 to 60 years, divided randomly into groups of 30 each, of American Society of Anesthesiologists (ASA) physical status classification I and II, who were put for elective surgical procedures to be done under general anaesthesia during the period of August 2018 to July 2020, were accepted. Patients were given 0.6 mg kg-1 IV of rocuronium in Group R and 0.15 mg kg-1 IV of cisatracurium in Group C. After injecting the muscle relaxants, parameters were measured 60, 90, 120, 150, 180, 240, and 300 seconds later.

Sample size

Openepi.com was used to carry out the sample size calculation considering the onset of action at 86.66±28.62 seconds (Meena et al. [9]) keeping power at 80% and confidence intervals at 95% (alpha error at 0.05); a sample of 26 patients in each group would be required to detect a difference of at least 30% in the onset of action among the two study groups. Considering the probable dropouts, the total sample size was kept at 60, i.e., 30 samples in each group.

Study criteria

*Inclusion Criteria* 

For surgical procedures to be performed under general anaesthesia, a sample of patients of ASA physical status I and II, within the age group 20 to 60 years, weight 40-80 kg, Mallampati pre-anaesthesia evaluation class I and II, who willingly gave informed consent, was included.

*Exclusion Criteria* 

Patients who did not consent, patients with respiratory problems or increased risk of pulmonary aspiration, renal disease, hepatic disease, cardiovascular and neuromuscular disease, patients under anticonvulsant, calcium channel blockers, β-blockers, steroids, anti-diuretics or aminoglycoside therapy, and those who were morbidly obese with anticipated difficult intubation were excluded.

Anaesthesia technique

*General Anaesthesia* 

Pre-anaesthetic checkup of physical evaluation, laboratory investigations regarding biochemistry, X-ray chest posteroanterior (PA) view, and electrocardiography (ECG) were performed and recorded, the day before the elective procedure. A fasting protocol of eight hours was maintained for all the patients. On the day of the procedure, IV access was obtained and IV fluids at the rate of 8 ml/kg body weight were infused through an 18G IV cannula in the preoperative room. Patients were premedicated with IV injections of pantoprazole 40mg and ondansetron 4mg. After shifting the patients to the operation theatre, haemodynamic variables including baseline ECG, peripheral oxygen saturation (SpO2), respiration rate, temperature, pulse rate, and blood pressure were checked and recorded. Surface warming was applied to maintain normothermia. After pre-oxygenation of the patients for three minutes, glycopyrrolate 0.004 mg kg-1, 0.05 mg kg-1 of midazolam and injection butorphanol 40 μg kg-1, all intravenously were given as pre-medicating agents. Anaesthesia was induced with injection propofol at a dose of 2 mg kg-1. An anaesthesiologist who was not in the intra-operating monitoring team prepared the drug solutions in stipulated pre-marked 5 ml syringes to avoid bias. The muscle relaxants administered were rocuronium bromide (Group R) 0.6 mg kg-1 IV and cisatracurium (Group C) 0.15 mg kg-1 IV over five seconds through rapidly running fluid infusion.

The investigator who was not informed of which drug out of the two relaxants was used, recorded the haemodynamic variables as heart rate (HR), systolic blood pressure (SBP), diastolic blood pressure (DBP) and compared the data at 60,90,120,150,180,240 and 300 seconds after injecting the muscle relaxants. The period of study was 300 seconds.

Onset is the period of time from the muscle relaxant injected to adequate jaw relaxation. The endotracheal tube of suitable size (7-7.5 ID for females, 8-8.5 ID for males) was used for intubation at a predetermined interval of 90 seconds after confirming adequate jaw relaxation by a skilled and experienced anaesthesiologist. Intubation was attempted only when the intubating circumstances/conditions were suitable (excellent or good), as per the Cooper et al. scale [10]. If the intubation was unsuccessful, it was tried again every 30 seconds.

The four-point scale of Cooper et al. (Table 1) states that the intubating conditions are graded as excellent, good, fair, and poor when the score is 8-9, 6-7, 3-5 or 0-2, respectively [10]. 

**Table 1:** Intubating conditions Evaluation and comparison of the intubating conditions in both groups were assessed according to the four-point scale of Cooper et al. [10].

Score	Jaw relaxation	Vocal cords	Response
0	Impossible to open	Closed	Coughing, bucking
1	Opens with difficulty	Closing	Mild cough
2	Moderate opening	Moving	Slight diaphragmatic movement
3	Easy opening	Open	None

During laryngoscopy, the intubating statuses in both the groups, as per vocal cord position and diaphragmatic movement/coughing, were assessed as excellent, good, and poor according to Fuchs‐Buder et al. (Table 2) [11].

**Table 2:** Intubating status assessment During laryngoscopy, the intubating status in both the groups, as per vocal cord position and diaphragmatic movement/coughing was assessed as excellent, good, or poor according to Fuchs‐Buder et al. [11].

Variable	Excellent	Good	Poor
Laryngoscopy	Easy	Fair	Difficult
Vocal cord position	Abducted	Intermediate/moving	Closed
Reaction as diaphragmatic movement: slight/vigorous/sustained contraction, &/or coughing for more than five seconds (>5s) due to insertion of the tracheal tube and cuff inflation	None	Slight (1-2 weak contractions for <5s)	Vigorous (>2 movements for >5s/coughing)

Once the endotracheal tube was checked after intubation for equal bilateral breath sound (on auscultation bilaterally: apices of the lungs 2 cm superior to medial one-third of clavicle; superior lobes - second intercostal space, midclavicular line, and between C7 and T3 posteriorly; middle lobe - fourth intercostal space, midclavicular line; inferior lobes - sixth intercostal space, mid-axillary line, and between T3 and T10 posteriorly) and end-tidal carbon dioxide (EtCO2) and then secured, anaesthesia was maintained using a closed breathing circuit and an intermittent maintenance dose of either of the muscle relaxants, 50% N2O in O2 and sevoflurane to a total 1.5%. Reversal of neuromuscular block was done by using injection glycopyrrolate 0.004 mg kg-1, neostigmine 0.05 mg kg-1, titrated according to response once the procedure was over and extubated when the patient regained consciousness with adequate muscle power. 

Statistical analysis

The software products IBM SPSS Statistics for Windows, Version 20, (Released 2011; IBM Corp., Armonk, New York, United States) and GraphPad Prism 6.0 version (GraphPad Software Inc., La Jolla, California, United States) were used in the analysis of the data; p <0.05 was considered statistically significant and highly significant if <0.001. Quantitative data were expressed as mean ± standard deviation (SD) and analyzed by independent sample t-test, while qualitative data were expressed as numbers and percentages and were analyzed by Chi-Square test (χ2).

## Results

As per our study the age, gender, and ASA physical status were comparable between the groups (Table 3).

**Table 3:** Demographic variables SD: Standard deviation; NS: Non-significant; ASA: American Society of Anesthesiologists

Variables	Cisatracurium	Rocuronium	p-value	Chi-Square test
Age (years) (mean ± SD)	40.56 ± 13.50	39.56 ±13.11	p = 0.77 NS	t = 0.2893
Gender (male/female)	15/15	16/14	p = 0.79 NS	χ^2^= 0.0667
ASA physical status classification I/II	14/16	15/15	p = 0.79 NS	χ^2^ = 0.0667

Our study shows the onset time (seconds) in rocuronium was 92 ± 7.61, while in cisatracurium, it was 188 ± 40.88 (p < 0.00001); the comparison is significant (Table 4). 

**Table 4:** Comparison of onset of action M: Mean; SD: Standard deviation

Study group	Onset (M)	Onset ( ± SD)	t value	p-value
Cisatracurium	188	± 40.88	12.64	˂ 0.00001
Rocuronium	92	± 7.61		

As per our observation, the patients in the rocuronium group had excellent to good intubating status (93.33%) at 90 seconds (Table 5). 

**Table 5:** Intubating status at different time intervals Fuchs-Buder et al. [11]

Intubating status	Different time intervals in both groups											
	90 seconds		120 seconds		150 seconds		180 seconds		240 seconds		300 seconds	
	C %	R %	C %	R %	C %	R %	C %	R %	C %	R %	C %	R. %
Excellent	-	22 73.33	-	2 6.66	2 6.66	-	4 13.33	-	8 26.66	-	4 13.33	-
Good	-	6 20	1 3.33	-	6 20	-	1 3.33	-	2 6.66	-	2 6.66	-
Poor	4 13.33	2 6.66	3 10	-	2 6.66	-	4 13.33	-	3 10	-	-	-
Inadequate	26 86.66	0	26 86.66	-	19 63.33	-	11 36.66	-	3 10	-	-	-
Intubated	0	28	1	2	8	0	5	0	10	0	6	0

We observed that intubating conditions of clinical acceptability occurred earlier in group R at 90 seconds (Table 6). 

**Table 6:** Intubating conditions at different time intervals Cooper et al. score [10] SD: Standard deviation; S: Significant

Time (seconds)	Cisatracurium (mean ± SD)	Rocuronium (mean ± SD)	t value	p-value
90	1.76 ± 0.77	7.8 ± 1.24	22.62	˂ 0.00001, S
120	2.56 ± 1.30	8.7 ± 0.70	22.73	˂ 0.00001, S
150	4.4 ± 1.95	9 ± 0	12.85	˂ 0.00001, S
180	6.43 ± 2.02	9 ± 0	6.93	˂ 0.00001, S
240	7.9 ± 1.76	9 ± 0	3.40	0.002, S
300	8.76 ± 0.56	9 ± 0	2.20	0.037, S

The difference between the mean heart rates of the two groups was statistically non-significant (p > 0.05) throughout our study period (Table 7). 

**Table 7:** Heart rate changes over time

TIime (seconds)	Cisatracurium (mean ± SD)	Rocuronium (mean ± SD)	t value	p-value
Baseline	75.70 ± 6.35	73.76 ± 6.45	1.16	0.24
60	78.66 ± 6.34	77.73± 6.46	0.56	0.57
90	85.66 ± 6.54	83.76 ± 6.59	1.11	0.26
120	84.53 ± 6.45	82.60 ± 6.47	1.15	0.25
150	83.50 ± 6.38	81.53 ± 6.37	1.19	0.23
180	81.60 ± 6.51	79.70 ± 6.49	1.13	0.26
240	78.70 ± 6.57	78.73 ± 6.65	0.01	0.98
300	77.5667 ± 6.53	76.60 ± 6.65	0.56	0.57

Slightly non-significant increases in systolic blood pressure (SBP) values were observed at 90 seconds in both C and R groups (p > 0.05) post laryngoscopy (Table 8).

**Table 8:** Systolic blood pressure (SBP) changes over time SD: Standard deviation

Time (seconds)	Cisatracurium (mean ± SD)	Rocuronium (mean ± SD)	t value	p-value
Baseline	122.30 ± 11.32	124.46 ± 10.33	0.77	0.44
60	126.30 ± 11.11	127.36 ± 10.35	0.38	0.70
90	133.80 ± 10.83	132.63 ± 10.22	0.43	0.66
120	134.10 ± 10.50	132.83 ± 9.93	0.48	0.63
150	131.56 ± 10.88	129.73 ± 9.78	0.68	0.49
180	127.56 ± 11.06	130.33 ± 9.80	1.02	0.31
240	125.50 ± 11.23	129.73 ± 9.43	1.57	0.12
300	123.53 ± 11.19	123.36 ± 9.71	0.05	0.95

In our study, following the administration of muscle relaxants, there were non-significant increases in both the groups' diastolic blood pressures observed at 90 seconds post laryngoscopy (Table 9).

**Table 9:** Diastolic blood pressure (DBP) changes over time SD: Standard deviation

Time (seconds)	Cisatracurium (mean ± SD)	Rocuronium (mean ± SD)	t value	p-value
BASELINE	78.06 ± 6.79	77.20 ± 6.12	0.52	0.60
60	79.76 ± 6.85	82.33 ± 6.21	1.51	0.13
90	83.73 ± 6.52	85.10 ± 6.32	0.82	0.41
120	83.70 ± 6.48	84.86 ± 6.06	0.72	0.47
150	82.83 ± 6.36	82.13 ± 6.11	0.43	0.66
180	82.96 ± 6.36	80.26 ± 6.24	1.65	0.10
240	81.56± 6.32	79.36 ± 6.04	1.37	0.17
300	79.90 ± 6.53	78.50 ± 5.97	0.86	0.39

## Discussion

The interval of time from the suppression of protective airway reflexes up to successful endotracheal intubation is a crucial period during the induction of GA because irreversible catastrophes including regurgitation and transbronchial aspiration of stomach contents can occur [12]. The degree of muscle relaxation, the depth of anaesthesia, and the ability of the anaesthesiologist are the criteria that determine how easily one can execute an intubation within the trachea. As long as rocuronium was not introduced into clinical practice, the search for the appropriate non-depolarizing drug for quick and secure endotracheal intubation persisted and continued until rocuronium was applied in therapeutic contexts. Thus, the current study was designed to examine the time of onset, intubating conditions and status, haemodynamic variable, and any unfavourable effects, for a comparison between the non-depolarizing drugs rocuronium (0.6 mg kg-1 IV), a 2 morpholino 3-diacetyl 16 N-allyl pyrrolidino derivative of vecuronium and cisatracurium (0.15 mg kg-1 IV), an isomer of NMBA atracurium, among 60 patients, 30 in each group. According to our data, the demographic criteria were non-significant (Table 3) between the groups.

Among the nondepolarizing neuromuscular blocking drugs, rocuronium has the fastest onset time [13]. Cisatracurium, a contemporary iso-quinolone NMBA with a similar advantage of Hoffmann degradation to laudanosine, a delayed onset, a moderate duration of action that is three to four times more effective than atracurium, does have less desirable intubating effects [14]. When compared to the effects of an equipotent dose of another non-depolarizing neuromuscular blocking drug, our decision to use rocuronium at a dose of 0.6 mg/kg and cisatracurium at a dose of 0.15 mg/kg was based on research done by other authors, who discovered that most children, adults, and elderly patients respond to a bolus dose of rocuronium at 0.6 mg/kg within 60-90 seconds to a clinically acceptable intubating condition [15]. In a study, it was found that using cisatracurium at different doses of 0.15 mg/kg (3 × ED95) and 0.2 mg/kg (4 × ED95) as part of an oxygen/nitrous oxide/propofol induction led to good or outstanding intubation conditions in 2.0 and 1.5 minutes, respectively.

A high-potency drug like cisatracurium undergoes buffered diffusion, which causes recurrent binding and unbinding to receptors, so the onset of action takes a longer time [16]. Drugs with low potency having less binding capacity to receptors, prevent buffered diffusion and increase receptor occupancy, resulting in fast onset of action. Hence, the time of onset of action in rocuronium is much shorter than that of cisatracurium. On the other hand, the time of onset for rocuronium was 0.7 minutes as found in one study [17]. It was observed that the onset time for the rocuronium group was 134 seconds versus 220 seconds for the cisatracurium group at 3 × ED95 dose and 95 seconds for the rocuronium group versus 162 seconds for the cisatracurium group at 4 × ED95 dose, according to a randomized controlled study between rocuronium and cisatracurium in equipotent doses [18].

The study by Adamus et al. [19] used the same dosages of rocuronium which was used in the previous reference [18]. The research by Adamus et al. on 120 patients found that the onset time for cisatracurium (0.10 or 0.15 mg kg(-1) or rocuronium (0.60 or 0.90 mg kg(-1) was roughly three times longer for CIS than for ROC for elective surgery with TIVA (total intravenous anaesthesia) but under tracheal intubation [19]. The same dose of rocuronium, 0.6mg/kg, was employed in children and adults by a number of authors, particularly when a peripheral vein was used for administration and a fast onset was needed, such as in rapid sequence intubation [20,21]. Taneja et al. observed that rocuronium 0.6 mg/kg dose had a considerably faster onset time than cisatracurium at 0.1 mg/kg when used as an intubating dose [22]. The onset time observed by us (Table 4) in rocuronium was 92 ± 7.61 seconds, while in cisatracurium, it was 188 ± 40.88 seconds (p < 0.00001), which is consistent with the findings of the above studies.

We used the four-point Cooper et al. grading scale (Table 1) for intubation conditions where grades were given as excellent, good, fair and poor when scores were 8-9, 6-7, 3-5, and 0-2, respectively [10]. 

Three factors (Table 2), according to Fuchs‐Buder et al. [11], consisting of laryngoscopy, vocal cord position, and diaphragmatic movement/coughing, were examined in both the groups in order to evaluate the intubating status in our study.

The data were then compiled. In our investigation, the intubating status was evaluated for the entire 300-second study period (Table 5), laryngoscopy was rated as easy in 24 patients (80%) in Group R and 16 patients in Group C (53.33%). Laryngoscopy was rated fair in 6 (20%) of Group R patients and 14 (46.66%) of Group C cases. Vocal cord position, such as whether they were abducted, intermediate or closed, as well as their movement during intubation, was documented. The vocal cords were abducted for 29 (96.66%) of Group C intubations (n=30) and in every intubation (100%) of Group R (n=30). Only 1 (3.33%) of Group C intubations resulted in vocal cord movement. The intubation score (Table 6) in our investigation according to Cooper et al. at 90 seconds was 1.76 ± 0.77 vs. 7.8 ± 1.24 in group C vs. group R, which was statistically significant (p < 0.00001). By 120 seconds, there was a difference between groups C and R of 2.56 ± 1.30 vs. 8.7 ± 0.70 (p < 0.00001), which was statistically significant. The score, which was 4.4 ± 1.95 vs. 9 ± 0 in groups C vs. R, was also significant (p <0.00001) by 150 seconds. The value of the Cooper et al. score at 180 seconds was 6.43 ± 2.02 vs. 9 ± 0 (p <0.00001) for groups C vs. R. At 240 and 300 seconds, the scores were 7.9 ± 1.76 vs. 9 ± 0 and 8.76 ± 0.56 vs. 9 ± 0 for groups C vs. R (p < 0.5), respectively (Table 6).

Thus, we observed that intubating conditions of clinical acceptability occurred earlier in group R of the two groups, which was 90 seconds. A study by Gupta et al. comparing the effects of rocuronium and vecuronium on intubating conditions, haemodynamic parameters, and commencement of action in 60 adult patients found that in the vecuronium group, 100% of patients receiving rocuronium had excellent intubating conditions. They discovered that rocuronium delivered clinically acceptable intubation conditions far sooner than vecuronium did at equipotent dosages. In the rocuronium group, all patients had good intubating conditions at 120 seconds, while 73.33% of patients had acceptable intubating conditions at 60 seconds [23]. In the study conducted by us, in the rocuronium group, we observed that 93.33% of the patients had satisfactory (excellent to good) intubating conditions at 90 seconds and all of the patients by 120 seconds (Table 5). 

Pino et al. observed at 90 seconds that only 40% of intubations with rocuronium were satisfactory [24], whereas our study showed 93.33% (Table 5) of patients had excellent to good intubation conditions.

Regarding haemodynamic parameter changes, we noticed that the baseline heart rate values (Table 7) in both groups (75.70 ± 6.35 and 73.76 ± 6.45 in the C and R groups, respectively) were comparable. The difference between the mean heart rates of the two groups was statistically non-significant (p > 0.05) across the groups throughout our study period. The heart rate started declining after 90 seconds and at 300 seconds (77.56 ± 6.53 in group C vs. 76.60 ± 6.65 in group R), heart rate values were closer to the baseline (Table 7). In the studies of Booth et al., the heart rate increased by 36% in the first minute after rocuronium injection [25]. The rise in our study was 25% (Table 7) at 60 seconds (one minute) from the baseline value.

A non-significant increase in SBP values, which can be attributed to pressor response, was observed at 90 seconds (133.80 ± 10.83 and 132.63 ± 10.22 in the C and R groups, respectively, p > 0.05) post laryngoscopy (Table 8). This increase from the baseline value may have been brought on by the mild vagolytic activity of rocuronium and the histamine release brought on by cisatracurium, as well as the stress response brought on by laryngoscopy and intubation. Following administration of the muscle relaxant, in our study, there was a negligible increase in both the groups' systolic and diastolic blood pressures (Tables 8, 9) observed at 90 seconds post laryngoscopy (p > 0.05). Our overall observation showed that the drugs rocuronium and cisatracurium are both stable haemodynamically. In our investigation, none of the groups showed indication of any substantial clinical cardiovascular abnormalities. 

When it comes to adverse effects, NMBAs can produce skin erythema at low doses and systemic vasodilation with hypotension and a rise in bronchomotor tone at high concentrations [16,26]. Numerous investigations have also documented a safe profile clinically with no side effects when rocuronium and cisatracurium are used [27,28]. No patient in our study group displayed any symptoms like bronchospasm, urticaria, wheal or cutaneous flushing associated with histamine release.

Thus, as per the observations of our study results, the intubating conditions of favourable/clinical acceptability as well as the onset time (seconds) of action was the earliest in rocuronium bromide (group R) in comparison to cisatracurium besylate (group C) with nonsignificant changes in haemodynamic status.

Limitation

A small sample size and heterogeneity of the population were there in our study; besides, the duration of action of the muscle relaxants, as well as patients of extreme age and patients with co-morbidities, were not studied. The effect of the muscle relaxant was not measured using a ToFscan accelerometer (Dräger, Lübeck, Germany) during the onset and recovery of neuromuscular blockade. Our study was not carried out with the muscle relaxants in different (ED95) doses. Another limitation of our study was that we only used butorphanol and propofol as premedication and induction agents, thus the findings cannot be predicted to other drugs and/or doses. We did not experience severe bradycardia or hypotension during this study performed on ASA I or II patients, but induction with propofol in critically ill patients can induce severe hypotension, so the drug needs to be used with caution in such patients. The bispectral index (BIS) was also not monitored to assess awareness. A comparative study with other intermediate action non-depolarizing drugs could have been carried out.

## Conclusions

The observation in our study conducted in elective patients for surgery under general anaesthesia, for comparison of two non-depolarizing NMBAs of intermediate duration of action, correlating the variables such as the degree of jaw relaxation and vocal cord immobility, was that rocuronium bromide (Group R) had good to excellent intubating conditions by 90 seconds, cisatracurium besylate (Group C) by 240 seconds, and the onset of action in Group R was at 92 ± 7.61 seconds and in Group C at 188 ± 40.88 seconds. Both are potent and safe having stable haemodynamic status with no adverse effect.
